# Supplementary material for: Investigation of the genetic aetiology of Lewy body diseases with and without dementia
Source: medRxiv. 2023 Oct 27:2023.10.17.23297157. Preprint. [Version 2] doi: 10.1101/2023.10.17.23297157 (PMC10659505; doi:10.1101/2023.10.17.23297157)
Supplement: Supplement 1 [file media-1.pdf]

Supplementary Table 1. Cohort description

Cohort	Abbreviation	N	Genotyping array
Tracking Parkinson’s Disease	TDP	2000	Illumina HumanCoreExome array
Oxford Parkinson's Disease Centre Discovery Cohort	OPDC	1082	Illumina HumanCoreExome-12 v1.1 or Illumina Infinium HumanCoreExome-24 v1.1
BioFIND	BF	213	Whole Genome Sequenced
Harvard Biomarker Study	HB	1173	Whole Genome Sequenced
Lewy Body Dementia Study	LB	4579	Whole Genome Sequenced
LRRK2 Cohort Consortium	LC	599	Whole Genome Sequenced
Parkinson’s Disease Biomarkers Program	PD	1604	Whole Genome Sequenced
Parkinson’s Progression Markers Initiative	PP	1943	Whole Genome Sequenced
Steady PD - Phase 3	SY	329	Whole Genome Sequenced
Sure PD - Phase 3	SU	259	Whole Genome Sequenced

Supplementary Table 2. Allele frequency

Groups		Rs17442721	Rs34637584 (*LRRK2 G2019S*)
LRRK2 carriers	LBD	45%	45%
	Non LBD	47%	46%
Non LRRK2 carriers	LBD	2%	0%
	Controls*	2%	0%

*Data extracted from non-Finnish Europeans in gnomAD browser (https://gnomad.broadinstitute.org/variant/12-40141971-C-G?dataset=gnomad_r3)

Supplementary Table 3. Dementia in LRRK2 G20219S carriers

Groups	LBD-D	LBD-ND
Patients without *LRRK2 G2019S*	2893 (39%)	4593 (61%)
Patients with *LRRK2 G2019S*	15 (5%)	303 (95%)

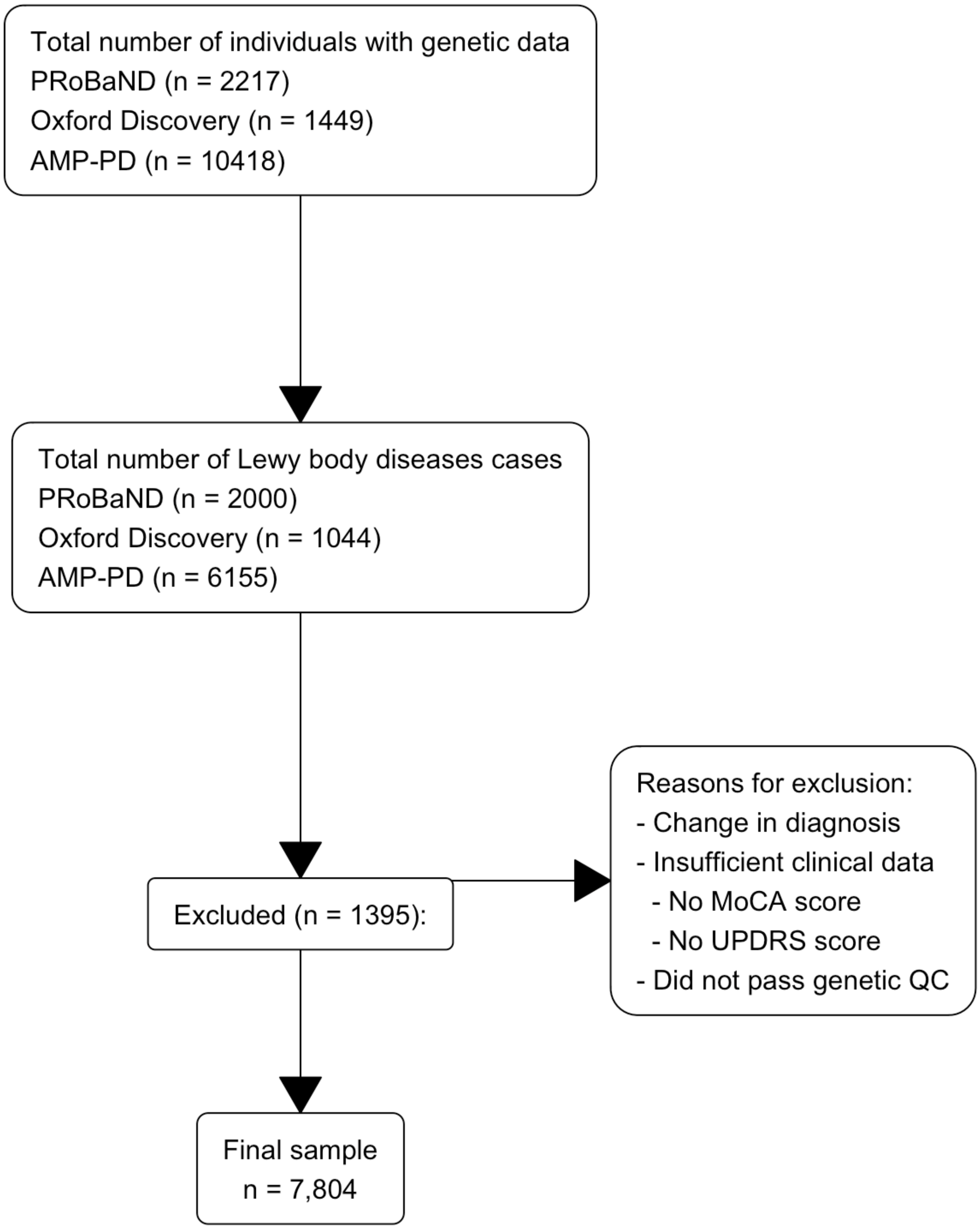

Supplementary figure 1. Sample selection workflow

Samples were selected based on their diagnosis, and whether they passed clinical and genetic data quality control.

1)

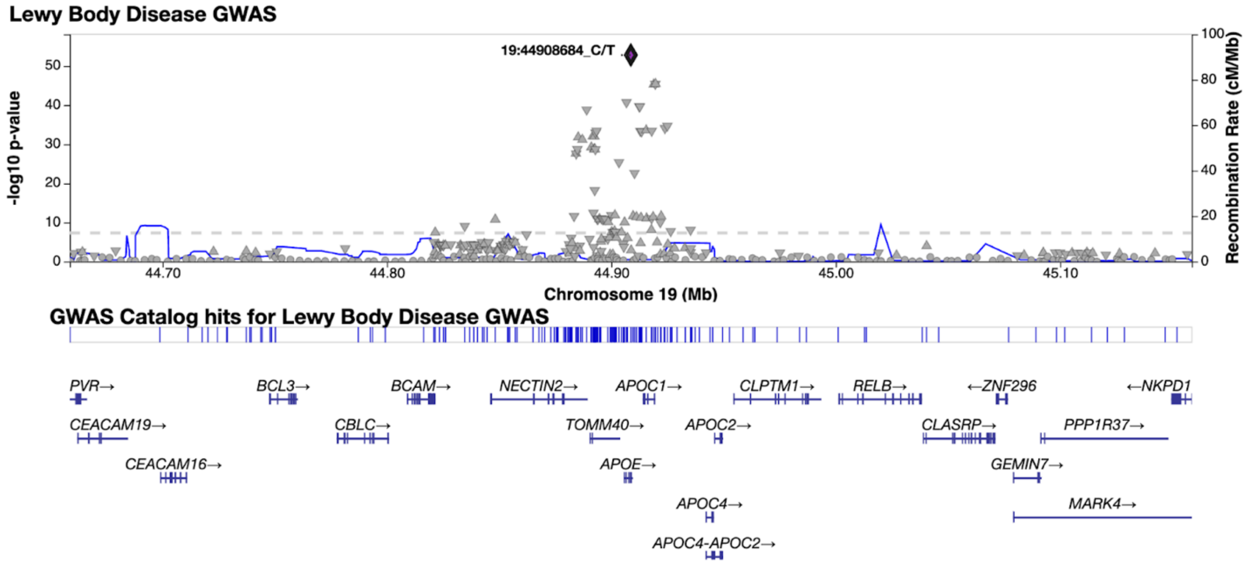
Lewy Body Disease GWAS
19:44908684_C/T
-log10 p-value
Recombination Rate (cM/Mb)
Chromosome 19 (Mb)
GWAS Catalog hits for Lewy Body Disease GWAS
PVR→
CEACAM19→
CEACAM16→
BCL3→
CBLC→
BCAM→
NECTIN2→
TOMM40→
APOE→
APOC1→
APOC2→
APOC4→
APOC4-APOC2→
CLPTM1→
RELB→
←ZNF296
CLASRP→
GEMIN7→
MARK4→
←NKPD1
PPP1R37→

2)

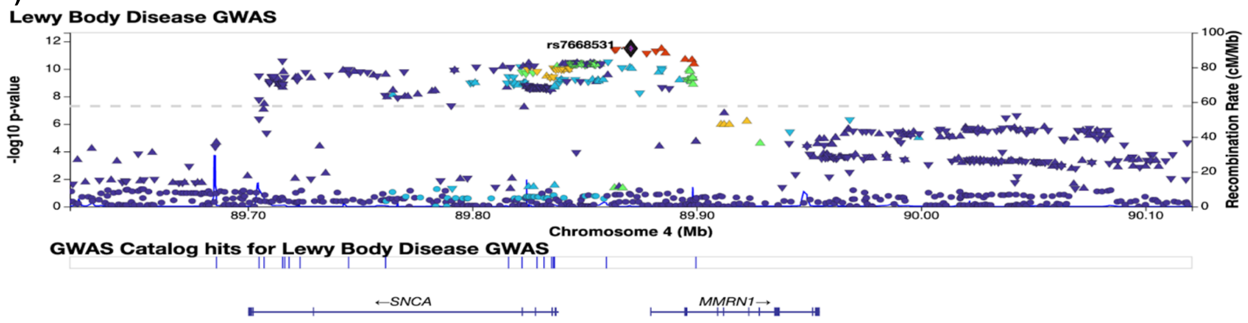
Lewy Body Disease GWAS
rs7668531
-log10 p-value
Recombination Rate (cM/Mb)
Chromosome 4 (Mb)
GWAS Catalog hits for Lewy Body Disease GWAS
←SNCA
MMRN1→

3)

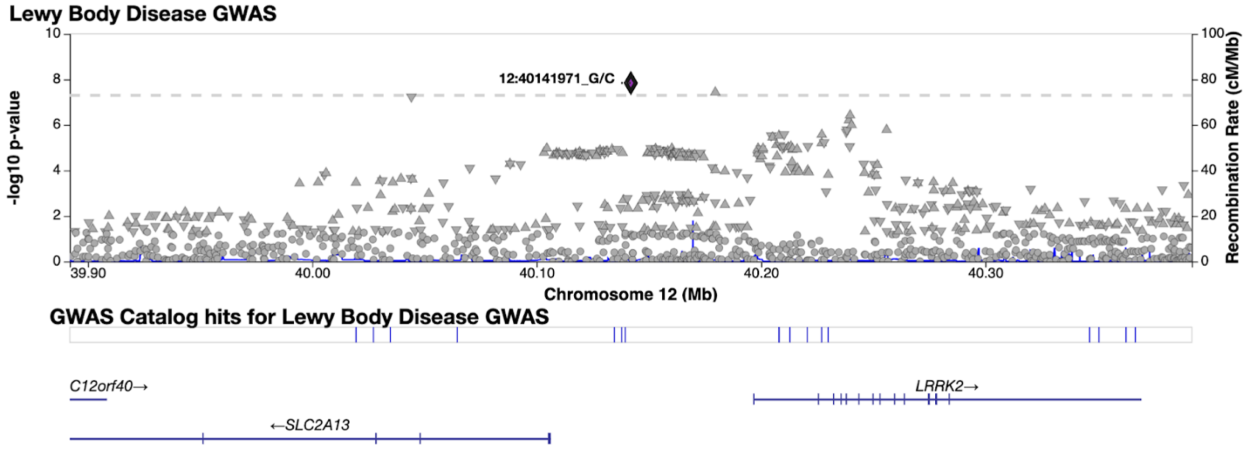

Supplementary Figure 2: Regional association plots of genome-wide significant SNPs

Regional association plots for 1) 19:44908684 (rs429358), 2) 4:89870668 (rs7668531) and 3) 12:40141971 (rs17442721). SNP position and recombination rates are based on GRCh38.

Appendix

International LBD Genomics Consortium (iLBDGC)

United States

- **Yevgeniya Abramzon, B.Sc.** (Neuromuscular Diseases Research Section, Laboratory of Neurogenetics, National Institute on Aging, Bethesda, MD, 20892, USA)
- **Sarah Ahmed, B.Sc.** (Neurodegenerative Diseases Research Unit, Laboratory of Neurogenetics, National Institute of Neurological Disorders and Stroke, Bethesda, MD, 20892, USA)
- **Camille Alba, Ph.D.** (Department of Anatomy, Physiology and Genetics, Physiology and Genetics, Uniformed Services University of the Health Sciences, Bethesda, MD, 20814, USA)
- **Marilyn S. Albert, Ph.D.** (Department of Neurology, Johns Hopkins University Medical Center, Baltimore, MD, 21287, USA)
- **Dagmar Bacikova, Ph.D.** (Department of Anatomy, Physiology and Genetics, Physiology and Genetics, Uniformed Services University of the Health Sciences, Bethesda, MD, 20814, USA)
- **Matthew J. Barrett, M.D.** (Department of Neurology, University of Virginia School of Medicine, Charlottesville, VA, 22903, USA)
- **Thomas G. Beach, M.D., Ph.D.** (Civin Laboratory for Neuropathology, Banner Sun Health Research Institute, Sun City, AZ, 85006, USA)
- **David A. Bennett, M.D.** (Rush Alzheimer's Disease Center, Rush University, Chicago, IL, 60612, USA)
- **Lilah M. Besser, Ph.D., M.Ph.** (Institute for Human Health and Disease Intervention, Florida Atlantic University, Boca Raton, FL, 33431, USA)

- **Eileen H. Bigio, M.D.** (Mesulam Center for Cognitive Neurology and Alzheimer's Disease, Northwestern University Feinberg School of Medicine, Chicago, IL, 60611, USA)
- **Bradley F. Boeve, M.D.** (Center for Sleep Medicine, Mayo Clinic, Rochester, MN, 55905, USA)
- **Ryan C. Bohannan, B.Sc.** (Department of Neurobiology and Behavior, University of California Irvine, Irvine, CA, 92697, USA)
- **Chad A. Caraway, Ph.D.** (Institute for Memory Impairments and Neurological Disorders, University of California Irvine, Irvine, CA, 92697, USA)
- **Jose-Alberto Palma, M.D., Ph.D.** (Department of Neurology, New York University School of Medicine, New York, NY, 10016, USA)
- **Ruth Chia, Ph.D.** (Neuromuscular Diseases Research Section, Laboratory of Neurogenetics, National Institute on Aging, Bethesda, MD, 20892, USA)
- **Clifton L. Dalgard, Ph.D.** (Department of Anatomy, Physiology and Genetics, Uniformed Services University of the Health Sciences, Bethesda, MD, 20814, USA; The American Genome Center, Collaborative Health Initiative Research Program, Uniformed Services University of the Health Sciences, Bethesda, MD, 20814, USA)
- **Dennis Dickson, M.D.** (Department of Neuroscience, Mayo Clinic, Jacksonville, FL, 32224, USA)
- **Jinhui Ding, Ph.D.** (Computational Biology Core, Laboratory of Neurogenetics, National Institute on Aging, Bethesda, MD, 20892, USA)
- **Kelley Faber, M.Sc.** (Department of Medical and Molecular Genetics, Indiana University School of Medicine, Indianapolis, IN, 46202, USA)
- **Tanis Ferman, Ph.D.** (Department of Psychiatry and Psychology, Mayo Clinic, Jacksonville, FL, 32224, USA)
- **Luigi Ferrucci, M.D., Ph.D.** (Longitudinal Studies Section, National Institute on Aging, Baltimore, MD, 21224, USA)
- **Margaret E. Flanagan, M.D.** (Northwestern University Feinberg School of Medicine, Chicago, IL, 60611, USA)
- **Tatiana M. Foroud, M.D.** (Department of Medical and Molecular Genetics, Indiana University School of Medicine, Indianapolis, IN, 46202, USA)
- **Bernardino Ghetti, M.D.** (Department of Pathology and Laboratory Medicine, Indiana University School of Medicine, Indianapolis, IN, 46202, USA)
- **J. Raphael Gibbs, Ph.D.** (Computational Biology Core, Laboratory of Neurogenetics, National Institute on Aging, Bethesda, MD, 20892, USA)
- **Alison Goate, Ph.D.** (Nash Family Department of Neuroscience, Department of Genetics and Genomic Sciences, and Department of Pathology, Icahn School of Medicine at Mount Sinai, New York, NY, 10029, USA)
- **David Goldstein, M.D.** (Clinical Neurocardiology Section, National Institute of Neurological Disorders and Stroke, Bethesda, MD, 20892, USA)
- **Neill R. Graff-Radford, M.D.** (Department of Neurology, Mayo Clinic Florida, 4500 San Pablo Road South, Jacksonville, FL, 32224, USA)
- **Heng-Chen Hu, Ph.D.** (Department of Anatomy, Physiology and Genetics, Physiology and Genetics, Uniformed Services University of the Health Sciences, Bethesda, MD, 20814, USA)
- **Daniel Hupalo, Ph.D.** (Department of Anatomy, Physiology and Genetics, Physiology and Genetics, Uniformed Services University of the Health Sciences, Bethesda, MD, 20814, USA)
- **Scott M. Kaiser, M.B.A.** (Department of Neuropathology, Indiana University School of Medicine, Indianapolis, IN, 46202, USA)

- **Horacio Kaufmann, M.D.** (Department of Neurology, New York University School of Medicine, New York, NY, 10016, USA)
- **Ronald C. Kim, M.D.** (Department of Neuropathology, School of Medicine, University of California Irvine, Irvine, CA, 92697, USA)
- **Gregory Klein** (Rush Alzheimer's Disease Center, Rush University, Chicago, IL, 60612, USA)
- **Walter Kukull, Ph.D.** (National Alzheimer's Coordinating Center (NACC), University of Washington, Seattle, WA, 98195, USA)
- **Amanda Kuzma, M.Sc.** (Department of Pathology and Laboratory Medicine, Perelman School of Medicine, University of Pennsylvania, Philadelphia, PA, USA)
- **James Leverenz, M.D.** (Cleveland Lou Ruvo Center for Brain Health, Neurological Institute, Cleveland Clinic, Cleveland, OH, 44195, USA)
- **Grisel Lopez, M.D.** (Medical Genetics Branch, National Human Genome Research Institute, Bethesda, MD, 20892, USA)
- **Qinwen Mao, M.D., Ph.D.** (Northwestern University Feinberg School of Medicine, Chicago, IL, 60611, USA)
- **Elisa Martinez-McGrath, Ph.D.** (Department of Anatomy, Physiology and Genetics, Physiology and Genetics, Uniformed Services University of the Health Sciences, Bethesda, MD, 20814, USA)
- **Eliezer Masliah, M.D.** (Molecular Neuropathology Section, Laboratory of Neurogenetics, National Institute on Aging, Bethesda, MD, 20892, USA)
- **Ed Monuki, M.D., Ph.D.** (Department of Pathology & Laboratory Medicine, School of Medicine, University of California Irvine, Irvine, CA, 92697, USA)
- **Kathy L. Newell, M.D.** (Department of Pathology and Laboratory Medicine, University of Kansas Medical Center, Kansas City, KS, 66160, USA)
- **Lucy Norcliffe-Kaufmann, Ph.D.** (Department of Neurology, New York University School of Medicine, New York, NY, 10016, USA)
- **Matthew Perkins, B.Sc.** (Michigan Brain Bank, University of Michigan Medical School, Ann Arbor, MI, 48109, USA)
- **Olga Pletnikova, M.D.** (Department of Pathology [Neuropathology], Johns Hopkins University Medical Center, Baltimore, MD, 21287, USA)
- **Alan E. Renton, Ph.D.** (Department of Neuroscience, Icahn School of Medicine at Mount Sinai, New York, NY, 10029, USA)
- **Susan M. Resnick, M.D.** (Laboratory of Behavioral Neuroscience, National Institute on Aging, Baltimore, MD, 21224, USA)
- **Owen A. Ross, Ph.D.** (Department of Neuroscience & Department of Clinical Genomics, Mayo Clinic Florida, 4500 San Pablo Road South, Jacksonville, FL, 32224, USA)
- **Marya S. Sabir, B.Sc.** (Neurodegenerative Diseases Research Unit, Laboratory of Neurogenetics, National Institute of Neurological Disorders and Stroke, Bethesda, MD, 20892, USA)
- **Clemens R. Scherzer, M.D.** (Precision Neurology Program, Brigham & Women's Hospital, Harvard Medical School, Boston, MA, 02115, USA)
- **Sonja W. Scholz, M.D., Ph.D.** (Neurodegenerative Diseases Research Unit, Laboratory of Neurogenetics, National Institute of Neurological Disorders and Stroke, Bethesda, MD, 20892, USA; Department of Neurology, Johns Hopkins University Medical Center, Baltimore, MD, 21287, USA)
- **Geidy Serrano, Ph.D.** (Civin Laboratory for Neuropathology, Banner Sun Health Research Institute, Sun City, AZ, 85006, USA)
- **Vikram Shakkotai, M.D., Ph.D.** (Department of Neurology, University of Michigan Medical School, Ann Arbor, MI, 48109, USA)

- **Ellen Sidransky, M.D.** (Medical Genetics Branch, National Human Genome Research Institute, Bethesda, MD, 20892, USA)
- **Andrew B. Singleton, Ph.D.** (Molecular Genetics Section, Laboratory of Neurogenetics, National Institute on Aging, Bethesda, MD, 20892, USA)
- **Toshiko Tanaka, Ph.D.** (Longitudinal Studies Section, National Institute on Aging, Baltimore, MD, 21224, USA)
- **Nahid Tayebi, Ph.D.** (Medical Genetics Branch, National Human Genome Research Institute, Bethesda, MD, 20892, USA)
- **Bryan J. Traynor, M.D., Ph.D.** (Neuromuscular Diseases Research Section, Laboratory of Neurogenetics, National Institute on Aging, Bethesda, MD, 20892, USA; Department of Neurology, Johns Hopkins University Medical Center, Baltimore, MD, 21287, USA)
- **Juan C. Troncoso, M.D.** (Department of Pathology [Neuropathology], Johns Hopkins University Medical Center, Baltimore, MD, 21287, USA)
- **Coralie Viollet, Ph.D.** (Department of Anatomy, Physiology and Genetics, Physiology and Genetics, Uniformed Services University of the Health Sciences, Bethesda, MD, 20814, USA)
- **Ronald L. Walton, B.Sc.** (Department of Neuroscience, Mayo Clinic Florida, Jacksonville, FL, 32224, USA)
- **Randy Woltjer, M.D., Ph.D.** (Department of Neurology, Oregon Health & Sciences University, Portland, OR, 97239, USA)
- **Zbigniew K. Wszolek, M.D.** (Department of Neurology, Mayo Clinic Florida, 4500 San Pablo Road South, Jacksonville, FL, 32224, USA)

Canada

- **Sandra E. Black, M.D.** (Institute of Medical Science, Faculty of Medicine, University of Toronto, 1 King’s College Circle, Room 2374, Toronto, ON, M5S 1A8, Canada; Division of Neurology, Department of Medicine, University of Toronto, 27 King's College Circle, Toronto, ON, M5S 1A1, Canada; Heart and Stroke Foundation Canadian Partnership for Stroke Recovery, Sunnybrook Health Sciences Centre, University of Toronto, 1 King’s College Circle, Room 2374, Toronto, ON, M5S 1A8, Canada; Hurvitz Brain Sciences Research Program, Sunnybrook Research Institute, University of Toronto, 2075 Bayview Avenue, Toronto, ON, M4N 3M5, Canada; LC Campbell Cognitive Neurology Research Unit, Sunnybrook Research Institute, University of Toronto, 2075 Bayview Avenue, Toronto, ON, M4N 3M5, Canada)
- **Ziv Gan-Or, M.D., Ph.D.** (Montreal Neurological Institute and Hospital, Department of Neurology & Neurosurgery, McGill University, 3801 University Street, Montreal, QC, H3A 2B4, Canada)
- **Julia Keith, M.D.** (Department of Anatomical Pathology, Sunnybrook Health Sciences Centre, University of Toronto, 1 King’s College Circle, Room 2374, Toronto, ON, M5S 1A8, Canada)
- **Mario Masellis, M.D., Ph.D.** (Cognitive & Movement Disorders Clinic, Sunnybrook Health Sciences Centre, University of Toronto, 1 King’s College Circle, Room 2374, Toronto, ON, M5S 1A8, Canada; Department of Medicine, Division of Neurology, University of Toronto, Toronto, ON, M5S 1A8, Canada; Hurvitz Brain Sciences Research Program, Sunnybrook Research Institute, University of Toronto, Toronto, ON, M5S 1A8, Canada; LC Campbell Cognitive Neurology Research Unit, Sunnybrook Research Institute, University of Toronto, Toronto, ON, M5S 1A8, Canada)
- **Ekaterina Rogaeva, Ph.D.** (Tanz Centre for Research in Neurodegenerative Diseases, University of Toronto, 1 King's College Circle, Room 2111, Toronto, ON, M5S 1A8, Canada)

United Kingdom

- **Dag Aarsland, M.D.** (Department of Old Age Psychiatry, Institute of Psychiatry, Psychology and Neuroscience [IoPPN], King's College London, DeCrespigny Park, London, SE5 8AF, UK)
- **Safa Al-Sarraj, M.D., Ph.D.** (Department of Clinical Neuropathology and London Neurodegenerative Diseases Brain Bank, Institute of Psychiatry, Psychology and Neuroscience [IoPPN], King's College Hospital and King's College London, DeCrespigny Park, London, SE5 8AF, UK)
- **Johannes Attems, M.D.** (Mental Health, Dementia and Neurodegeneration, Translational and Clinical Research Institute, Newcastle University, Newcastle upon Tyne, NE4 5PL, UK)
- **Raffaele Ferrari, Ph.D.** (Department of Molecular Neuroscience, Institute of Neurology, University College London, London, WC1B 5EH, UK)
- **Steve Gentleman, M.D.** (Neuropathology Unit, Division of Brain Sciences, Department of Medicine, Imperial College London, London, W12 0NN, UK)
- **John A. Hardy, Ph.D.** (Department of Neurodegenerative Disease, Reta Lila Weston Laboratories, Queen Square Genomics, UCL Dementia Research Institute, London WC1E 6BT, UK)
- **Angela K. Hodges, Ph.D.** (Department of Old Age Psychiatry, Institute of Psychiatry, Psychology and Neuroscience [IoPPN], King's College London, Maurice Wohl Clinical Neuroscience Institute, London, SE5 9NU, UK)
- **Seth Love, M.D.** (Dementia Research Group, School of Clinical Sciences, University of Bristol, Bristol, BS10 5NB, UK)
- **Ian McKeith, M.D.** (Mental Health, Dementia and Neurodegeneration, Translational and Clinical Research Institute, Newcastle University, Newcastle upon Tyne, NE4 5PL, UK)
- **Christopher M. Morris, Ph.D.** (Mental Health, Dementia and Neurodegeneration, Translational and Clinical Research Institute, Newcastle University, Newcastle upon Tyne, NE4 5PL, UK)
- **Huw R. Morris, M.D., Ph.D.** (Department of Molecular Neuroscience, Institute of Neurology, University College London, London, WC1B 5EH, UK; Department of Clinical and Movement Neuroscience, Royal Free Campus UCL Institute of Neurology, University College London, London, NW3 2PF, UK)
- **Laura Palmer, Ph.D.** (South West Dementia Brain Bank, Bristol Medical School, University of Bristol, Bristol, BS10 5NB, UK)
- **Stuart Pickering-Brown, Ph.D.** (Division of Neuroscience and Experimental Psychology, Faculty of Biology, Medicine and Health, The University of Manchester, Manchester, M13 9PT, UK)
- **Regina H. Reynolds, M.Sc.** (NIHR Great Ormond Street Hospital Biomedical Research Centre, University College London, London, UK; Genetics and Genomic Medicine, Great Ormond Street Institute of Child Health, University College London, London WC1E 6BT, UK; Department of Neurodegenerative Disease, UCL Queen Square Institute of Neurology, University College London, London, UK)
- **Mina Ryten, M.D., Ph.D.** (NIHR Great Ormond Street Hospital Biomedical Research Centre, University College London, London, UK; Genetics and Genomic Medicine, Great Ormond Street Institute of Child Health, University College London, London WC1E 6BT, UK)
- **Alan J. Thomas, M.B.B.S., Ph.D.** (Mental Health, Dementia and Neurodegeneration, Translational and Clinical Research Institute, Newcastle University, Newcastle upon Tyne, NE4 5PL, UK)

- **Bension S. Tilley, M.B.B.S., Ph.D.** (Neuropathology Unit, Division of Brain Sciences, Department of Medicine, Imperial College London, London, W12 0NN, UK)
- **Claire Troakes, Ph.D.** (Department of Old Age Psychiatry, Department of Basic and Clinical Neuroscience, King's College London, DeCrespigny Park, London, SE5 8AF, UK)

Republic of Ireland

- **Francesca Brett, M.D.** (Dublin Brain Bank, Neuropathology Department, Beaumont Hospital, Dublin, D2, Ireland)

France

- **Alexis Brice, M.D.** (Paris Brain Institute, Paris Brain Institute, Sorbonne Universites, 47, boulevard de l'Hôpital, Paris, CS 21 414 – 75646, France)
- **Charles Duyckaerts, M.D.** (Paris Brain Institute, Paris Brain Institute, Sorbonne Universites, 47, boulevard de l'Hôpital, Paris, CS 21 414 – 75646, France)
- **Suzanne Lesage, Ph.D.** (Paris Brain Institute, ICM, Sorbonne Universites, 47, boulevard de l'Hôpital, Paris, CS 21 414 – 75646, France)

Italy

- **Maura Brunetti, M.Sc.** (Rita Levi Montalcini Department of Neuroscience, University of Turin, Turin, 10126, Italy)
- **Andrea Calvo, M.D.** (Rita Levi Montalcini Department of Neuroscience, University of Turin, Turin, 10126, Italy)
- **Antonio Canosa, M.D.** (Rita Levi Montalcini Department of Neuroscience, University of Turin, Turin, 10126, Italy)
- **Adriano Chiò, M.D.** (Rita Levi Montalcini Department of Neuroscience, University of Turin, Turin, 10126, Italy; Institute of Cognitive Sciences and Technologies, C.N.R., Via S. Martino della Battaglia, 44, Rome, 00185, Italy, Azienda Ospedaliero Universitaria Città della Salute e della Scienza, Corso Bramante, 88, Turin, 10126, Italy)
- **Gianluca Floris, M.D.** (Department of Neurology, University Hospital of Cagliari, Via Ospedale, 54, Cagliari, 09124, Italy)
- **Giancarlo Logroscino, M.D., Ph.D.** (Department of Basic Medical Sciences, Neurosciences and Sense Organs, University of Bari "Aldo Moro", Bari, 70121, Italy)
- **Chiara Zecca, B.Sc.** (Department of Basic Medical Sciences, Neurosciences and Sense Organs, University of Bari "Aldo Moro", Bari, 70121, Italy)
- **Jordi Clarimon, Ph.D.** (Universitat Autònoma de Barcelona, Carrer de Sant Quintí, 77-79, Barcelona, 08041, Spain, and Movement Disorders Units, Department of Neurology, University Hospital Mutua de Terrassa, Barcelona, 08221, Spain)
- **Monica Diez-Fairen, M.Sc.** (Memory and Movement Disorders Units, Department of Neurology, University Hospital Mutua de Terrassa, 08221 Barcelona, Spain)
- **Juan Fortea, M.D., Ph.D.** (Universitat Autònoma de Barcelona, Carrer de Sant Quintí, 77-79, Barcelona, 08041, Spain, and Movement Disorders Units, Department of Neurology, University Hospital Mutua de Terrassa, Barcelona, 08221, Spain)
- **Isabel González-Aramburu, M.D., Ph.D.** (Neurology Service, University Hospital Marqués de Valdecilla-IDIVAL-UC, Santander, 39011, Spain)
- **Jon Infante, M.D., Ph.D.** (Neurology Service, University Hospital Marqués de Valdecilla-IDIVAL-UC, Santander, 39011, Spain)
- **Carmen Lage, M.D.** (Neurology Service, University Hospital Marqués de Valdecilla-IDIVAL-UC, Santander, 39011, Spain)

- **Alberto Lleó, M.D., Ph.D.** (Universitat Autònoma de Barcelona, Carrer de Sant Quintí, 77-79, Barcelona, 08041, Spain, and Movement Disorders Units, Department of Neurology, University Hospital Mutua de Terrassa, Barcelona, 08221, Spain)
- **Pau Pastor, M.D., Ph.D.** (Memory and Movement Disorders Units, Department of Neurology, University Hospital Mutua de Terrassa, 08221 Barcelona, Spain)
- **Laura Porcel-Molina, M.D., Ph.D.** (Neurological Tissue Bank, Biobanc-Hospital Clinic - IDIBAPS, C/ Centre Esther Koplowitz, Barcelona, 08036, Spain)
- **Eloy Rodríguez-Rodríguez, M.D., Ph.D.** (Neurology Service, University Hospital Marqués de Valdecilla-IDIVAL-UC, Santander, 39011, Spain)
- **Pascual Sanchez-Juan, M.D., Ph.D.** (Neurology Service, University Hospital Marqués de Valdecilla-IDIVAL-UC, Santander, 39011, Spain)

Luxembourg

- **Rejko Krüger, M.D.** (Luxembourg Center for Systems Biomedicine, University of Luxembourg, Esch-sur-Alzette, Luxembourg, L-4362, Luxembourg; Luxembourg Institute of Health (LIH), University of Luxembourg, Esch-sur-Alzette, Luxembourg, L-4362, Luxembourg; Centre Hospitalier de Luxembourg (CHL), University of Luxembourg, Esch-sur-Alzette, Luxembourg, L-4362, Luxembourg)
- **Patrick May, Ph.D.** (Luxembourg Center for Systems Biomedicine, University of Luxembourg, Esch-sur-Alzette, Luxembourg, L-4362, Luxembourg)

Greece

- **Georgia Xiromerisiou, M.D., Ph.D.** (Department of Neurology, University Hospital of Larissa, University of Thessalia, Mezourlo, Larissa, 41110, Greece)
